# Clients’ satisfaction with cervical cancer screening services and influencing factors at public health facilities in Debre Markos town, Northwest Ethiopia, 2022/23: a convergent parallel mixed method

**DOI:** 10.1186/s12905-024-03250-5

**Published:** 2024-08-02

**Authors:** Alemu Merga Hailu, Fisseha Yetwale Kassie, Beyene Sisay Damtew, Muhabaw Shumye Mihret

**Affiliations:** 1 Department of Midwifery, School of Nursing and Midwifery, Institute of Health Sciences, Wollega University, Nekemte, Ethiopia; 2 Department of Clinical Midwifery, School of Midwifery, College of Medicine and Health Sciences, University of Gondar, Gondar, Ethiopia; 3 Department of Midwifery, School of Nursing and Midwifery, College of Medicine and Health Sciences, Arsi University, Asella, Ethiopia

**Keywords:** Cervical cancer, Cervical cancer screening, Satisfaction, Mixed method, Debre Markos

## Abstract

**Background:**

Satisfaction is defined as the perceived fulfillment of patient or client needs and desires through the delivery of healthcare services. In developed countries, more than 60% of women have been screened for cervical cancer. However, only 12% of women in sub-Saharan Africa have been screened for precancerous cervical lesions. There is limited evidence on client satisfaction with cervical cancer screening services (CSCCSS) in Ethiopia, particularly, there is no study conducted by mixed method in the Amhara region.

**Objective:**

The study aimed to assess clients’ satisfaction with cervical cancer screening services and influencing factors among women screened in Debre Markos town public health facilities in Northwest Ethiopia, 2022/23.

**Methods:**

A convergent parallel mixed methods design was conducted in Debre Markos town’s public health facilities from October 10th, 2022 to January 10th, 2023. For the quantitative wing, a total of 401 cervical cancer screening service users were selected using a systematic random sampling technique. Data were collected using an interviewer-administered structured questionnaire. Clients were interviewed on exit in a private area far from the screening unit and the data were entered into Epi-data version 4.6.0.2, then exported to STATA version 14 for analysis. A binary logistic regression model was fitted to identify factors associated with client satisfaction with cervical cancer screening services. The qualitative data were collected through in-depth and key informant interviews using a semi-structured topic guide. The data were analyzed using a thematic analysis approach with Open code software (version 4.0.2.3).

**Result:**

The quantitative wing revealed that overall, 65% (95% CI: 60–69) of respondents were satisfied with the cervical cancer screening services they received. Gender of the provider (AOR: 6.11, 95% CI: 3.23–11.55, p-value = 0.000), waiting time (AOR: 4.77, 95% CI: 1.32–17.31, p-value = 0.017), clients’ knowledge (AOR: 0.26, 95% CI: 0.12–0.59, p-value = 0.001), and clients’ attitude (AOR: 6.43, 95% CI: 3.43–12.03, p-value = 0.000) were significantly associated with CSCCSS.

**Qualitative result:**

The thematic analysis revealed three themes. Theme 1: facility-related barriers (shortage of skilled manpower, shortage of infrastructure, providers’ skill gap, unavailability of full service, leadership problem, long waiting time). Theme 2: client-related barriers (poor knowledge and attitude, gender preference). Theme 3: facility-related facilitators (free service, presence of supportive partners).

**Conclusion:**

According to the findings of this study, two-thirds of clients were satisfied with cervical cancer screening services, which was lower than the national target of 80%. Long waiting time, male gender of the service provider, unfavorable attitude, and good knowledge of clients were identified as significant factors negatively affecting client satisfaction with cervical cancer screening.

## Introduction

Globally, an estimated 604,000 new cases and 342,000 deaths of cervical cancer were reported in 2020, with 80% of these cases coming from Low- and Middle-Income Countries (LMIC) [1]. Africa has the highest regional morbidity and mortality of cervical cancer, and Malawi has the highest rates [2]. In Ethiopia, cervical cancer is the second leading cause of cancer in women, with an estimated 7,400 new cases and 5,300 deaths reported in 2023 [3].

More than 99% of cervical cancer precursors are caused by a sexually transmitted virus called human papillomavirus (HPV) [4]. Although there are more than 200 HPV strains, most of cervical neoplasia caused by oncogenic strains, such as HPV-16, HPV-33, HPV-18, HPV-31, HPV-45, HPV-52, and HPV-58 [5]. But, the most invasive malignancies are predominantly caused by types 16 and 18 [6]. Fortunately, HPV infections induced cervical cancer is preventable by avoiding the risk factors, HPV vaccination and screening for precancerous lesion and treatment [7]. It is possible to manage non-invasive cervical lesions using a variety of effective methods. Despite the effectiveness of the therapeutic approaches, HPV still poses a risk of recurrence and persistence [8].

The World Health Organization (WHO) recommends that 70% of women globally should be regularly screened for cervical cancer with a high-performance test, and 90% of those needing it should receive appropriate treatment [9]. Visual inspection with acetic acid (VIA) is the most effective, acceptable, and affordable method to detect cervical cancer precursors and is widely used in under-resourced settings [10].

**Fig. 1:**
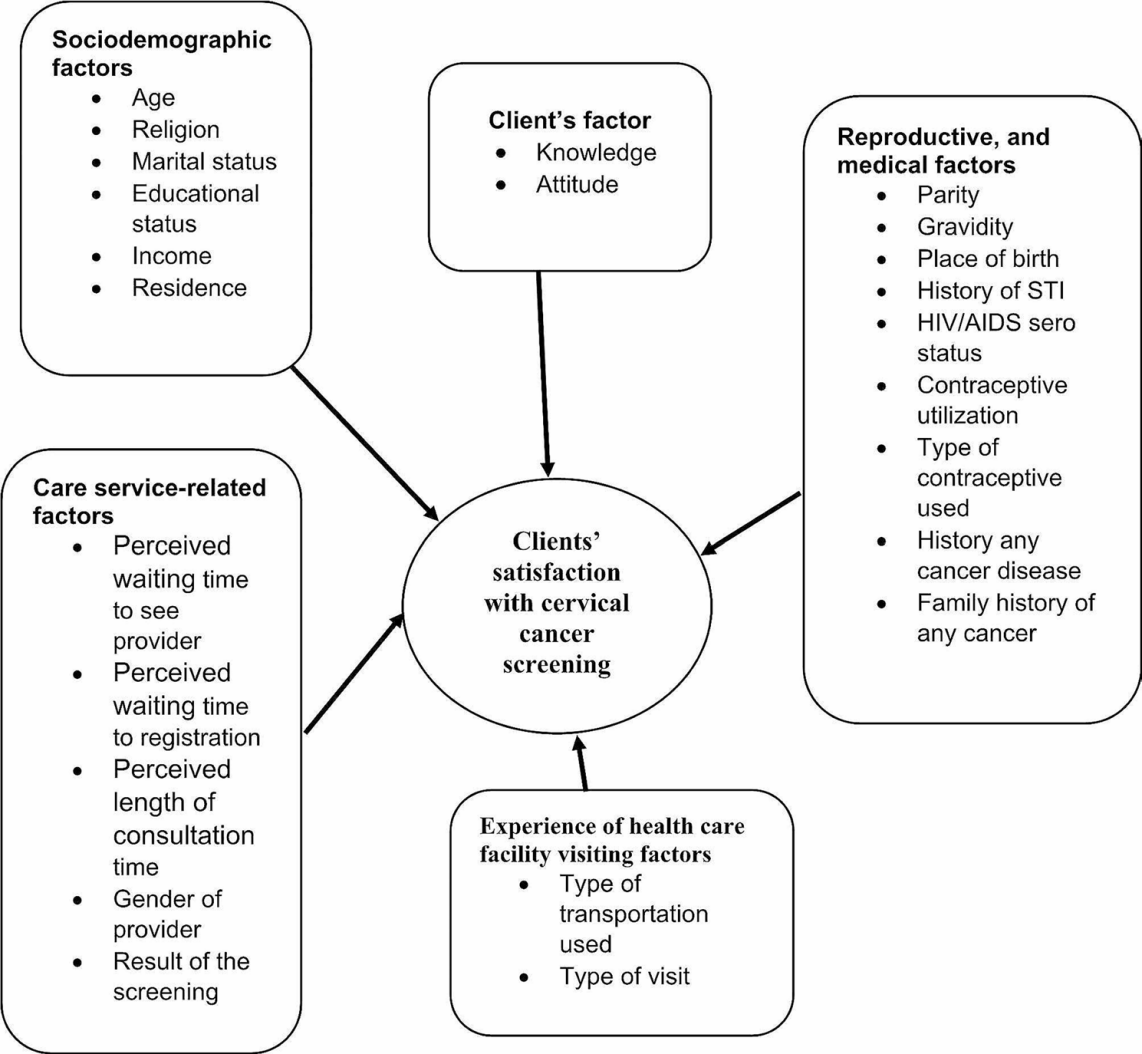
Conceptual framework showing contributing factors of client’s satisfaction with cervical cancer screening services as adapted from different literatures(24, 25, 27, 41, 44, 49, 50)

Findings from studies conducted in Thailand, Morocco and Malawi on the level of satisfaction of clients with CCS services shown that 59.2%, 97% and 100% of women received the services were satisfied respectively and waiting time, religion, knowledge, and attitude of clients, educational status and marital status were factors affecting satisfaction [11–13]. A similar study conducted in Ethiopia revealed that, 41% of women satisfied with the cervical cancer screening [14] Fig. 1.

Despite being preventable with vaccination and screening, cervical cancer has low uptake of screening in low and middle-income countries [15, 16]. Factors such as low-quality screening services and failure to widely implement the program contribute to this low utilization [17].

In health care, satisfaction is defined as the perceived fulfillment of patient or client needs and desires through the delivery of healthcare services [18]. Satisfaction with healthcare services is considered a good indicator of the effectiveness of the healthcare system [19]. Moreover, satisfaction with health care service is clinically essential for adhering to treatment instructions, client retention, and improving outcomes [20]. Enhancing the quality and client satisfaction of cervical cancer screening services (CSCCS) is a main strategy to improve the acceptance of screening services [21]. Studies show that low satisfaction with screening services is one of the main factors contributing to low utilization of cervical cancer screening services [22–24].

However, level of satisfaction with cervical cancer screening is not well known, particularly in low-income countries. WHO is striving to eliminate cervical cancer by adopting the Global Strategy for cervical cancer elimination (90-70-90 strategy) [25]. Ethiopia has also launched a national CCS program since 2015, aiming to screen women aged 30–49 to reduce the high incidence of cervical cancer [26].

In spite of all of these efforts, and the fact that many health care facilities began offering screening services following the programs, screening coverage remained low. Although several studies have been conducted on uptake and barriers to cervical cancer screening services, there was a scarcity of evidences about clients’ satisfaction with these services in Ethiopia particularly in Amhara region as to the investigators’ best knowledge. Moreover, deep rooted multi-perspective contexts related to CSCCSS remain less explored. In this regard, mixed methods research (MMR) is especially important in LMIC settings, where understanding social, economic and cultural contexts are essential to assess health systems performance [27].

A MMR study is being conducted to explore diverse perspectives and uncover relationships that exist between the intricate layers of multifaceted research questions. The results from this study will help in identifying areas that need more attention in CCS by addressing areas for improvement. Knowing the status of client satisfaction and identifying factors related to satisfaction with the service will help all stakeholders, including policymakers, healthcare institutions, health teaching institutions, and healthcare providers, in focusing more on the problem and developing strategies to address it. Additionally, the findings of the research will be used as a reference for other researchers.

## Materials and methods

### Study settings

The study was conducted in Debre Markos town, which is located 299 km from the capital city of the country, Addis Ababa, and 265 km from Bahir-Dar, the capital city of the Amhara Regional State. The town has a total population estimated to be 92,470, with 46,738 being females [28]. There is one comprehensive specialized hospital, four health centers, and more than ten private clinics in Debre Markos town. Currently, the comprehensive specialized hospital and three health centers are providing cervical cancer screening services.

### Study design, period, and population

A convergent parallel mixed method was conducted, from October 10th, 2022 to January 10th, 2023.

For the quantitative strand the source population consisted of all cervical cancer screening recipient 30–49 aged women in Debre Markos town public health facilities. All women who had received cervical cancer screening services in the selected health facilities during the data collection period and provided informed consent were included in the quantitative wing.

The qualitative aspect of the study included cervical cancer screening service users who did not participate in the quantitative part, the service providers who delivered the services for both categories of service users, and administrators in Debre Markos town.

### Sample size

Sample size was calculated using single population proportion formula (n = (Z_α/2_)^2^ p(1-p)/d^2^), by taking 41% the proportion of clients satisfied with cervical cancer screening service from previous study conducted in Jimma, Ethiopia [14]. So, the sample size was n= (1.96)^2^*0.41*(1-0.41)/(0.05)^2^= 372 by adding 10% nonresponse rate the final sample size was 410. Table 1.

**Table 1:** Showing list of significant variables to calculate sample size

Variables	Percent of outcome in unexposed	Power	CI	OR	Sample size with 10% non-response	References
Religion	46%	80%	95%	2.34	220	(27)
Perceived waiting of time	65%	80%	95%	2.18	294	(27)

### Sampling procedure

**Fig. 2:**
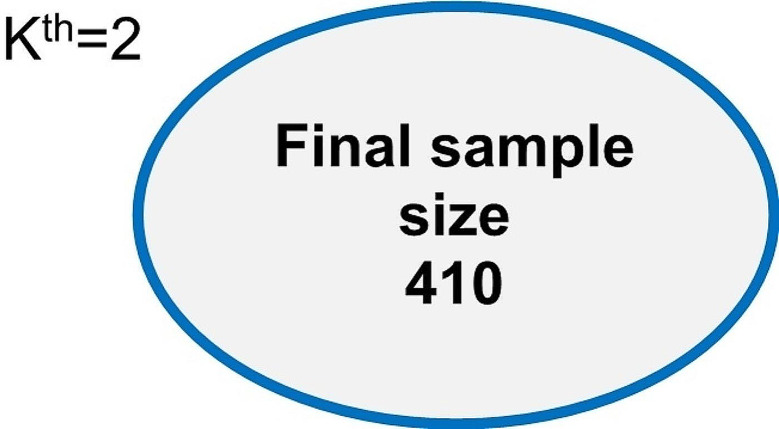
Schematic presentation of sampling technique for study client’s satisfaction with cervical cancer screening and associated factors among women screened for cervical cancer in Debre Markos public health facilities, North West Ethiopia, 2022/23

There are four public health institutions providing cervical cancer screening in Debre Markos town administration. All public cervical cancer screening centers in the town were selected. Sample size was allocated proportionally for each health facilities based on clients screened for cervical cancer in recent one quarter. The average clients screened in the last one quarter was 446, 165, 108, and 96 for Debre Markos Comprehensive Specialized Hospital, Debre Markos Health Center, Hidase Health Center and Wuseta Health Center respectively. The study participants were selected from source population by systematic random sampling. Based on a systematic random sampling methodology (i.e., Kth = N / sample size = > calculated for each facility$\:\approx\:$ 2 which means K^th^= 2), an assessment of the four Debre Markos town public health facilities average women screened was made. For the qualitative wing we were employed purposive sampling (Fig. 2).

### Operational definitions

Client satisfaction- Client or patient satisfaction is a psychological state that can be described as a patient’s reaction to the environment, process, and outcome of the healthcare service received [29].

Satisfied- Clients who scored 36 or higher [14, 30].

Dissatisfied- Clients who scored less than 36 [14, 30].

Good knowledge- Clients who scored greater or equal to the median score [12, 14].

Poor knowledge- Clients who scored less than the median score [12, 14].

Favorable Attitude- Clients who scored greater or equal to the mean score [9, 14].

Unfavorable Attitude- Clients who scored less than the mean score [9, 14].

Waiting time- was perceived time a client had spent at registration, after registration to see service provider and during consultation.

Overall waiting time was addition of waiting time at registration, after registration to see the service provider and consultation time. Based on the Business Process Re-engineering (BPR) waiting time calculation for outpatient departments of health facilities, waiting time of less than 120 min were considered as short waiting time, while waiting time greater than or equal to 120 min were considered long [31].

### Data collection tools and procedure the quantitative strand

Data were collected by using structured, pretested and face to face interviewer administered questionnaire. The data collection questionnaire was adapted from previous similar studies conducted at the country [14, 32–34]. Finally, a research expert was consulted and reviewed the tool. The questionnaire was initially prepared in English, translated into the local language Amharic, and then back to English by language experts to check its consistency. The Amharic version of the questionnaire was used for data collection. Data were collected by four trained healthcare providers. Clients were interviewed on exit in a private area far from the screening unit. The questionnaire contains satisfaction indicators related to sociodemographic characteristics of clients and different dimensions of the services, such as perceived waiting time, sex of the provider, and knowledge and attitude of clients to cervical cancer and the screening test, as well as satisfaction parameters.

#### Data quality control

To ensure consistency and improve clarity, the questionnaire was written in English and then translated into Amharic and back to English. To ascertain the reliability, clarity and appropriateness of the tool before the real data collection 5% pretest was done at Yejube Hospital. Training was given for the supervisors and data collectors for two days. The training was focused on the objective of the study, the contents of the questionnaire, how to collect the data, on issues related to the confidentiality of the response and the rights of the respondents. During data collection, the questionnaires were checked for completeness daily by the supervisors.

### Data collection tools and procedure for the qualitative strand

Data were collected through in-depth interviews (IDIs) and Key informant interviews (KIIs) using a semi-structured interview guideline. Data were collected using the Amharic language, and each interview was recorded with the participants’ permission in the health facilities. The data were collected by the investigator. The data collection tool contains basic information about the socio-demographic characteristics of the participants and topic guides for the IDIs and KIIs. The tool was initially prepared in English and finally translated to the local language Amharic. Interviews were conducted face-to-face individually with participants in a private room. The interviews with all participants were audiotaped, and field notes were taken.

#### Trustworthiness

Trustworthiness is a general term employed to express scientific rigor in qualitative research [35]. The central concept of trustworthiness is related to answering the question “Can the study be trusted?” Trustworthiness expresses the qualitative study’s truthfulness, applicability, consistency, and neutrality from the researcher’s bias [36].

#### Credibility

During the research process, we used peer debriefing to discuss the qualitative research with advisors and experienced individuals. We also conducted probing interviews to support our data collection.

#### Dependability

To ensure dependability, our study findings were reviewed by advisors and experienced researchers, and we made sure that our literature and transcripts were consistent with our results.

#### Transferability

For transferability, we provided a clear description of the entire research process, from data collection to final results reporting, and checked and corrected all audio-taped interviews and transcripts. Participant statements were quoted directly, and detailed definition was developed between the studied context and the participant’s statement.

#### Confirmability

To establish confirmability, we transcribed audio records and typed notes verbatim, and carefully analyzed the data for any personal bias.

### Data processing and analysis for the quantitative strand

After checking the completeness and accuracy of the collected data, a code was provided to the questionnaire, and data were entered Epi-data version 4.6.0.2 and then exported to STATA version 14 for analysis. Data cleaning were done to check for accuracy, consistencies and missed values. The clients’ satisfaction was assessed using 12 satisfaction items with a five-point Likert scale ranging from very dissatisfied [1] to very satisfied [5]. Clients were categorized in to two (satisfied and dissatisfied) based on level of their satisfaction using demarcation threshold formula- (Total highest score – Total lowest score)/2 + Total lowest score. Accordingly, the cutoff point was 36. Women who scored less than 36 were categorized as “Dissatisfied”, whereas, those who scored greater or equal to 36 were considered “Satisfied” [14].

Knowledge of clients were assessed by 23 general knowledge questions. 21 questions were delivered to the study participants with “yes” or “no” options and the left 2 questions were presented with multiple answers and then dichotomized to “yes” or “no” options. Each correct response was given a score of 1 and a wrong answer given a score of 0. We computed the sum of 23 scored items. Finally, we classified knowledge as poor and good by computing the median score to obtain the cutoff point. The median score was 12. The women who scored less than 12 were considered to have “poor knowledge” whereas those who scored greater or equal to 12 were considered to have “good knowledge“ [14].

Attitudes of clients were assessed using 12 items with a five-level Likert scale that contains “Very Disagree”, “Disagree”, “Neutral”, “Agree” and “Very Agree”. Then very disagree, disagree, and neutral answers were given a score of 0, and agree and very agree answers were given a score of 1. We computed the sum of 12 scored items. The mean score was calculated and found to be 9. Accordingly, Women who scored less than 9 were considered to have “unfavorable attitudes”, whereas those who scored greater than or equal to 9 were considered to have “favorable attitudes“ [14].

Both descriptive and analytical statistical procedures were performed to summarize the data. Tables were used to present the study results. Binary logistic regression model was used to find out independent predictors of client’s level of satisfaction with cervical cancer screening services.

The study involved both descriptive and analytical statistical procedures to summarize the data. The results were presented using tables. A binary logistic regression model was used to identify independent predictors of client satisfaction with cervical cancer screening services. Bivariable and multivariable logistic regression analyses were conducted to identify the explanatory variables significantly associated with the dependent variable. Both crude odds ratio (COR) and adjusted odds ratio (AOR) with the corresponding 95% confidence intervals (CI) were calculated. Variables with a p-value of less than 0.2 in binary logistic regression were included in the multivariable logistic regression to control for confounders. In multivariable logistic regression, a p-value of 0.05 with a 95% confidence interval was used to determine the level of statistical significance. Multicollinearity was checked using variance inflation factors (VIF), and all variables had values less than 10. The model fitness was assessed using Hosmer and Lemeshow’s goodness-of-fit test, resulting in a p-value of 0.849.

### Data processing and analysis the qualitative strand

**Fig. 3:**
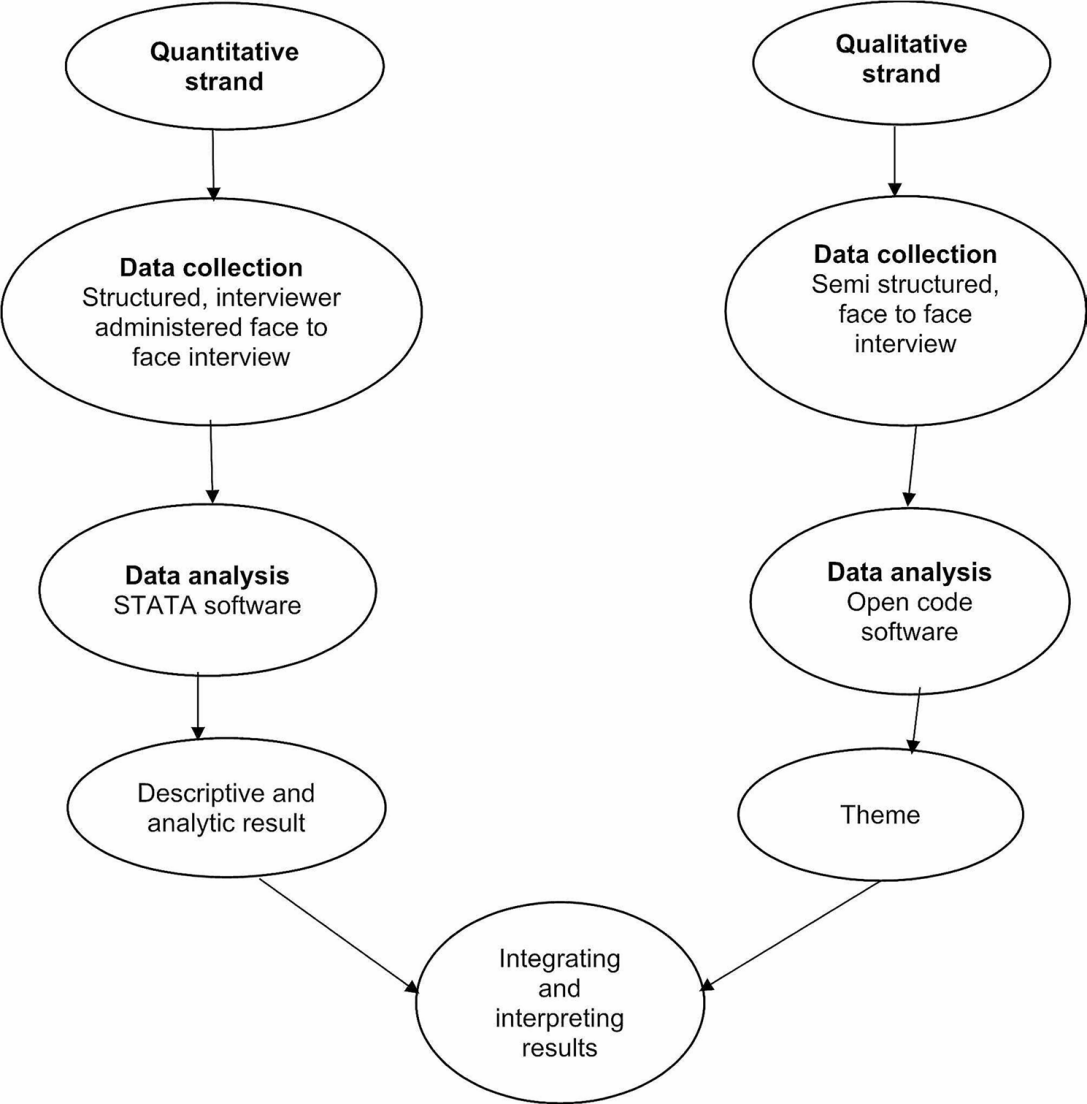
Flowchart of convergent parallel mixed method implemented in the study

For the qualitative strand, thematic analysis was used for data processing and analysis. The collected data were transcribed verbatim and translated back into English by language experts. The transcriptions and translations were reviewed several times to complete the coding process. The open code software (version 4.0.2.3) was utilized to manage the collected data, identifying important points raised throughout the interview and repeatedly mentioning information that addressed the research questions. The coding was repeated several times to merge similar codes and eliminate unsupported codes. Parental codes were selected and grouped under sub-themes based on similar concepts. Finally, themes were merged by combining several similar categories Fig. 3.

### Results for the quantitative strand

#### Socio demographic characteristics

A total of 401 cervical cancer screened 30–49 aged women were interviewed, yielding a response rate of 97.8%. The mean age of the respondents was 35.9 years, with 49% of the respondents aged 30–34 years. The majority of the women were married, followers of the Orthodox religion, and urban residents. Educational levels varied, with 36% having no formal education and 28% attending college and above. Additionally, 24% were government employees. Ninety-seven (24%) were government employees by occupation Table 2.

**Table 2:** Socio-demographic and economic characteristics of women, Debre Markos town, Northwest Ethiopia, 2023 (*n* = 401)

Variables	Frequency (*N* = 401)	Percent (%)
Age in years		
30–34	196	49
35–39	98	24
40–44	72	18
45–49	35	9
Residence		
Urban	275	69
Rural	126	31
Occupation		
Government employee	97	24
Housewife	82	21
Farmer	84	21
Merchant	93	23
Student	17	4
Daily laborer	16	4
Other	12	3
Educational level		
Cannot read and write	131	33
Can read and write but no formal education	11	3
Primary school (1-8th)	83	21
Secondary school (9-12th)	65	16
College and above	111	28
Religion		
Orthodox	378	94
Muslim	13	3
Protestant	10	3
Marital status		
Single	17	4
Married and live together	249	62
Married and live separately	40	10
Divorced	72	18
Widowed	23	6
Average estimated monthly household income (ETB)		
< 5000	231	58
5000–9999	121	30
10,000-14999	42	11
≥ 15,000	7	2

#### Reproductive and medical characteristics of the clients

Regarding reproductive and medical characteristics, most of the women were multiparous, 270(60%) and 96% had given birth at least once in a health facility. All study participants were tested for HIV/AIDS, with 38% testing positive. About 71% were screened for sexually transmitted infections (STIs), and 48% of them tested positive. Additionally, 9% had a family history of any cancer, and 2% had a self-history of cancer. Table 3.

**Table 3:** Reproductive and Medical characteristics of women, Debre Markos town, Northwest Ethiopia, 2023 (*n* = 401)

Variables	Frequency (*N* = 401)	Percent (%)
Pregnancy		
Ever been pregnant	360	90
Never been pregnant	41	10
Number of pregnancies		
1	80	22
2–4	215	60
≥ 5	65	18
Birth		
Ever gave birth	357	89
Never gave birth	44	11
Number of births		
1	87	25
2–4	216	60
≥ 5	54	15
Place of birth		
Home	13	4
Health facility	277	78
Both at home and health facility	63	18
HIV test		
Ever tested for HIV	401	100
HIV test result		
Positive	154	38
Negative	247	62
STI test		
Screened for STI	285	71
Never screened for STI	116	29
STI screen result		
Positive	137	48
Negative	148	52
History of cancer		
Have self-history of any cancer	7	2
Don’t have self-history of cancer	394	98
Have family history of any cancer	36	9
Don’t have family history of cancer	365	91
Modern contraceptive method utilization		
Ever used modern any contraceptive method	363	91
Never used modern contraceptive method	38	9
Type of contraceptive method used		
Pills	93	19
Injection	257	52
Implant	128	26
IUCD	3	1
Condom	12	2

#### Experiences of visiting health facility and characteristics of services received

Regarding reproductive and medical characteristics, most of the women were multiparous, 270(60%) and 96% had given birth at least once in a health facility. All study participants were tested for HIV/AIDS, with 38% testing positive. About 71% were screened for sexually transmitted infections (STIs), and 48% of them tested positive. Additionally, 9% had a family history of any cancer, and 2% had a self-history of cancer. Table 4.

**Table 4:** Experiences to visiting health facility and characteristics of services, Debre Markos town, Northwest Ethiopia, 2023 (*n* = 401)

Mechanisms of health facility visit		
On foot	42	11
By public transport	349	86
By private vehicle	10	3
Overall perceived waiting time		
Short	376	94
Long	25	6
How to visit this facility today		
By appointment for the screening	66	16
For another service	335	84
Gender of the provider		
Male	122	30
Female	279	70
Result of the screening test		
Negative	346	84
Positive	55	14

#### Knowledge of clients on cervical cancer and its screening

Over all 53% (95% (CI = 48–58) of respondents had good knowledge of cervical cancer. The participants were asked whether they know the main risk factors of cervical cancer and less than half (35%) had answered correctly. Similarly, 52(13%) of respondents claim they did not know a single symptom of cervical cancer. The common symptoms asked were; vaginal bleeding, post coital bleeding, offensive vaginal discharge, pain during sexual intercourse and pelvic pain.

Regarding treatment, 223(56%) of participant’s said cervical cancer is curable if treated early and 254(63%) of them had known at least one of the treatment modalities of cervical cancer. Most of the participants 367(92%) had known that cervical cancer is preventable and known at least one the preventive mechanism like; avoiding multiple sexual intercourse, avoiding smoking, vaccination and avoiding early sexual intercourse initiation. Concerning cervical cancer screening, nearly all (94%) of them know the frequency of the screening service and two third (66%) knows the eligibility criteria for cervical cancer screening with VIA. Table 5.

**Table 5:** Knowledge of clients on risk factors, symptoms, treatment, and prevention of cervical cancer, Debre Markos town, Northwest Ethiopia, 2023 (*n* = 401)

Variables	Yes (*n*, %)	No (*n*, %)
**Risk factors**		
Having multiple sexual partners is a risk factor for cervical cancer	330(82)	82 [18]
Having a previous family history of cervical cancer is a risk factor for individuals’ cervical cancer	130 [32]	271(68)
Smoking is a risk factor for cervical cancer	53 [13]	348(87)
STIs are a risk factor for cervical cancer	172(43)	229(57)
OCP use is a risk factor for cervical Cancer	28 [7]	373(93)
**Symptoms of cervical cancer**		
Early sexual intercourse is a risk factor to cervical cancer	238(59)	163 [41]
Cervical cancer has signs and symptoms	347(87)	54 [13]
Vaginal bleeding is symptom of cervical cancer	220(55)	181(45)
Post-coital vaginal bleeding is symptom of cervical cancer	194(48)	207(52)
Offensive vaginal discharge is symptom of cervical cancer	227(57)	174(43)
Painful coitus is symptom of cervical cancer	208(52)	193(48)
Pelvic pain is symptom of cervical cancer	227(57)	174(43)
**Treatment**		
Cancer of the cervix can be cured if treated at its earliest stages	223(56)	178(44)
Cervical cancer can be treated with surgery	69 [17]	332(83)
Cervical cancer can be treated with chemotherapy	169(42)	232(58)
Cervical cancer can be treated with Radiotherapy	134 [33]	267(67)
**Prevention**		
Avoiding multiple sexual partner	328(82)	73 [18]
Avoiding early sexual intercourse initiation	212(53)	189(47)
HPV vaccination	79 [20]	322(80)
Avoid smoking	56 [14]	345(86)
Screening for precancerous lesion	325(81)	76 [19]
Eligibility for cervical cancer screening	378(94)	23 [6]
Frequency of cervical cancer screening	265(66)	136 [34]

#### Attitude of clients towards cervical cancer and its screening

Over all 55% (95% (CI = 50–60) had favorable attitude of cervical cancer and screening. Majority, 345(86%) of participants strongly believed that its helpful to detect cervical cancer early. Only 107(27%) of the study participants strongly agree that they have a chance of getting cervical cancer any time in life, while 277(69%) have no idea. Only one fourth (20%) of them strongly agreed that any woman can acquire cervical cancer at any point of time. Almost one fifth (21%) of participants strongly agreed that cervical cancer can be treated. Table 6.

**Table 6:** Attitude of clients on cervical cancer and screening of cervical cancer, Debre Markos town, Northwest Ethiopia, 2023 (*n* = 401)

Attitude statements	Strongly disagree (*n*, %)	Disagree (*n*, %)	Neutral (*n*, %)	Agree (*n*, %)	Strongly agree (*n*, %)
It is helpful to detect cervical cancer early	4 [1]	6 [2]	9 [2]	37 [9]	345(86)
You have the chance of getting Cervical Cancer	73 [18]	95 [24]	98 [24]	28 [7]	107 [27]
Getting Cervical Cancer is a serious for you	3 [1]	0	5 [1]	27 [7]	366(91)
There are effective methods for cervical cancer prevention	4 [1]	14 [4]	52 [13]	65 [16]	266(66)
Carcinoma of the cervix can cause of death.	3 [1]	4 [1]	9 [2]	23 [6]	362(90)
Any women can acquire cervical cancer	105 [26]	125 [31]	75 [19]	16 [4]	80 [20]
Carcinoma of the cervix can be treated	99 [25]	110 [27]	70 [17]	38 [10]	84 [21]
Screening helps in prevention of cervical cancer	12 [3]	3 [1]	46 [11]	32 [8]	308(77)
You have Willingness of screening	1(0.25)	0	4 [1]	21 [5]	375(94)
You recommend cervical cancer screening for your friends, family and neighbors.	3 [1]	0	7 [2]	16 [4]	375(94)
Cervical cancer screening procedure is not too painful	64 [16]	53 [13]	125 [31]	37 [9]	122 [30]
The health professionals are respectful	6 [2]	10 [3]	29 [7]	28 [7]	328(81)

#### Satisfaction of clients with cervical cancer screening service

Overall, 260(65% (95% (CI = 60–69)) of respondents were satisfied with cervical cancer screening services they received. Regarding reception, 303(76%) of clients were strongly satisfied with the reception of health care professionals at cervical cancer screening unit. About 54% of interviewed clients were strongly satisfied with the cleanliness of the screening room, while 321(80) were strongly satisfied with the cleanliness of the screening equipment’s.

Among interviewed participants, 304(76%) were strongly satisfied with the waiting time to see providers and 295(74%) were strongly satisfied with the length of consultation time. Concerning privacy, nearly one third 133(33%) and 134(33%) of participated clients were strongly dissatisfied with auditory and visual privacy respectively. Nearly one fourth (23%) of respondents were strongly satisfied with the convenience of the physical examination. Regarding experience of the procedures, 54(14%) were strongly dissatisfied with the VIA screening. Table 7.

**Table 7:** Satisfaction of clients with cervical cancer screening services, in Debre Markos town public health facilities, Northwest Ethiopia, 2023 (*n* = 401)

Satisfaction statement	Strongly Dissatisfied (*n*, %)	Dissatisfied (*n*, %)	Neutral (*n*, %)	Satisfied (*n*, %)	Strongly satisfied (*n*, %)
Satisfaction with reception by providers	15 [4]	18 [4]	41 [10]	24 [6]	303(76)
Satisfaction with clarity of explanation on screening	73 [18]	58 [15]	44 [11]	77 [19]	149 [37]
Satisfaction with clarity of explanation on cervical cancer	81 [20]	54 [14]	45 [11]	87 [22]	134 [33]
Satisfaction with cleanliness of the screening room	98 [24]	31 [8]	27 [7]	29 [7]	216(54)
Satisfaction with waiting time to see provider	30 [7]	28 [7]	20 [5]	19 [5]	304(76)
Satisfaction with length of consultation time	9 [2]	21 [5]	41 [10]	35 [9]	295(74)
Satisfaction with convenience with physical examination	124 [31]	31 [8]	69 [17]	86 [21]	91 [23]
Satisfaction with cleanliness of the screening instruments	6 [2]	6 [2]	31 [7]	37 [9]	321(80)
Satisfaction with auditory privacy	133 [33]	18 [5]	45 [11]	45 [11]	160 [40]
Satisfaction with visual privacy	132 [33]	17 [4]	54 [13]	47 [12]	151 [38]
Satisfaction with self determination to be screened	4 [1]	12 [3]	74 [19]	33 [8]	278(69)
Satisfaction with VIA screening test experience	54 [14]	66 [16]	73 [18]	65 [16]	143 [36]

#### Factors associated with client’s satisfaction with cervical cancer screening service

Bivariable and Multivariable Binary Logistic Regression Analysis were done to assess factors associated with level of satisfaction of clients received cervical cancer screening services. All the independent variables were checked for multicollinearity and eligible variables had been fitted to the bivariable logistic regression. Variables with a *p* value of less than or equal to 0.2 (clients place of residence, occupation, level of education, religion, result of HIV test, STI test result, result of the cervical cancer screening, the way of visit, knowledge of clients, attitude of clients, age, gravidity, parity, perceived waiting time) were exported to the multivariable analysis to control the effect of possible confounders.

A p-value < 0.05 was used to declare a significant association in the multivariable binary logistic regression. Gender of provider, waiting time, knowledge and attitude of clients were significantly associated with client’s satisfaction with cervical cancer screening in the multivariable logistic regression. Table 8.

**Table 8:** Bivariable and multivariable logistic regression analysis of factors associated with client’s satisfaction with cervical cancer screening services in Debre Markos town public health facilities, Northwest Ethiopia, 2023 (*n* = 401)

Variables	Satisfied (*n*, %)	Dissatisfied (*n*, %)	COR (95% CI)	*p*-value	AOR (95% CI)	*p*-value
Age in years						
30–34	106 [41]	90(64)	1		1	
35–39	68 [26]	30 [21]	1.92(1.15–3.26)	0.012	1.09(0.56–2.25)	0.818
40–44	59 [23]	13 [9]	3.85(2.00-7.48)	0.000	2.40(0.92–6.29)	0.073
45–49	27 [10]	8 [6]	2.87(1.24–6.62)	0.014	1.66(0.43–6.39)	0.461
Residence						
Urban	162(62)	113(80)	1		1	
Rural	98 [38]	28 [20]	2.44(1.50–3.96)	0.000	0.57(0.19–1.76)	0.328
Occupation						
Housewife	53 [21]	29 [21]	1		1	
Government employee	48 [18]	49 [35]	0.54(0.29–0.98)	0.043	0.59(0.14–2.46)	0.468
Farmer	66 [25]	18 [13]	2.01(1.00–4.00)	0.048	1.25(0.38–4.05)	0.714
Merchant	69 [27]	24 [17]	1.57(0.82–3.01)	0.171	2.22(0.82–5.91)	0.116
Student	9 [3]	8 [6]	0.62(0.21–1.77)	0.367	2.21(0.42–10.76)	0.366
Daily laborer	9 [3]	7 [5]	0.70(0.24–2.09)	0.526	0.45(0.11–1.89)	0.276
Other	6 [2]	6 [4]	0.55(0.16–1.85)	0.332	0.82(0.14–4.73)	0.822
Educational level						
Cannot read and write	110(42)	21 [15]	1		1	
Can read and write but no formal education	8 [3]	3 [2]	0.51(0.12–2.08)	0.347	1.30(0.21–7.98)	0.776
Primary school (1-8th)	54 [21]	29 [21]	0.36(0.19–0.68)	0.002	0.85(0.33–2.17)	0.733
Secondary school (9-12th)	34 [13]	31 [22]	0.21(0.11–0.41)	0.000	0.40(0.13–1.26)	0.118
College and above	54 [21]	57 [40]	0.18(0.10–0.33)	0.000	0.50(0.11–2.19)	0.354
Religion						
Orthodox	249(96)	129(91)	1		1	
Muslim	6 [2]	7 [5]	0.44(0.15–1.35)	0.152	1.32(0.30–5.90)	0.713
Protestant	5 [2]	5 [4]	0.52(0.15–1.82)	0.305	0.97(0.16–5.90)	0.973
Number of pregnancies						
0	13 [5]	28 [20]	1		1	
1	39 [15]	41 [29]	2.05(0.93–4.52)	0.075	3.88(0.13–109.9)	0.433
2–4	157(60)	58 [41]	5.83(2.83-12.0)	0.000	3.26(0.08–137.1)	0.529
5 and above	51 [20]	14 [10]	7.85(2.24-19.0)	0.000	2.57(0.04–160.8)	0.646
Number of births						
0	15 [6]	29 [21]	1		1	
1	41 [16]	46 [32]	1.72(0.81–3.66)	0.156	0.53(0.02–14.46)	0.709
2–4	161(62)	55 [39]	5.66(2.83–11.3)	0.000	1.62(0.04–59.07)	0.792
5 and above	43 [16]	11 [8]	7.56(3.04–18.8)	0.000	2.04(0.04–114.2)	0.728
HIV test result						
Positive	116(45)	38 [27]	2.18(1.40–4.40)	0.001	1.52(0.82–2.84)	0.186
Negative	144(55)	103(73)	1		1	
STI test result						
Negative	102 [39]	46 [33]	1		1	
Positive	81 [31]	56 [41]	0.65(0.40–1.06)	0.086	0.68(0.34–1.36)	0.273
Unknown	77 [30]	39 [28]	0.89(1.53–1.50)	0.661	0.80(0.39–1.64)	0.544
Ever heard about cervical cancer						
Yes	125(48)	85(60)	0.61(0.40–0.92)	0.020	0.93(0.49–1.79)	0.831
No	135(52)	56 [40]	1		1	
Gender of the provider						
Male	51 [20]	71(50)	1		1	
Female	209(80)	70(50)	4.16(2.65–5.52)	0.000	6.11(3.23–11.55)	0.000
Mechanism of visiting the facility						
On foot	25 [10]	17 [12]	1		1	
By public transport	230(88)	119(84)	1.31(0.68–2.53)	0.113	1.89(0.71–5.06)	0.205
By private vehicle	5 [2]	5 [4]	0.68(0.17–2.71)	0.585	1.12(0.15–8.81)	0.915
Overall perceived waiting time						
Short	254(98)	122(87)	6.59(2.57–16.9)	0.000	4.77(1.32–17.31)	0.017
Long	6 [2]	19 [13]				
Result of cervical precancerous lesion screening						
Positive	45 [17]	10 [7]	2.74(1.34–5.62)	0.006	1.68(0.64–4.40)	0.294
Negative	215(83)	131(93)	1		1	
Knowledge						
Good	108(42)	103(73)	0.26(0.17–0.40)	0.000	0.26(0.12–0.59)	0.001
Poor	152(58)	38 [27]	1		1	
Attitude						
Favorable	174(67)	46 [33]	4.18(2.70–6.46)	0.000	6.43(3.43–12.03)	0.000
Unfavorable	86 [33]	95(67)	1		1	

Keys: AOR = Adjusted odd ratio, COR = Crude odd ratio, CI = Confidence interval, 1 = Reference category, STI- Sexual Transmitted infections, HIV- Human Immune Virus

As a result, clients received cervical cancer screening services from female health care providers were 6.11 times more satisfied with the services compared to those who received the service from female health care providers [AOR: 6.11,95% CI: (3.23–11.55), p-value = 0.000]. This is supported by the qualitative finding. The majority of clients participated in the qualitative part had reported that they prefer female provider to male. A major reason for their preference is the fact that they value privacy and freedom over everything else.

One of the clients interviewed said that; *“It is good that the provider was a female. I will not be ashamed of exposing my genitals to female health professionals*,* because we are the same nature. I am glad that I found a female provider. If I offered to choose*,* I would choose a female provider”. (*Participant#*11*,* 33 years old client)*

Another respondent noted that she prefers a female service provider to communicate freely and be screened without fear. *“I prefer a woman to be transparent*,* and examined freely. You can communicate freely because talking to another woman is not humiliating or frightening. This is my preference. I don’t know about others”. (*Participant#*I3*,* 33 years old client)*

Clients who reported shorter waiting time were 4.77 times more likely to be satisfied with cervical cancer screening services than those who reported longer waiting time [AOR: 4.77, 95% CI: (1.32–17.31), p-value = 0.017]. this is supported by the in-depth interview result of a 36 years old client “*I have been looking for the screening room for a long time. There were no labels on the room. When I asked people*,* I did not find anyone who told me and I have been looking for a long time. Upon finding the room*,* I did not find anyone inside. There was no one in the screening room. There were no health professionals. It was empty*,* so I sat down and waited*,* then he came. I waited for at least 25 minutes. So*,* they shouldn’t disappear from their workplace”.*

Women who had good knowledge of cervical cancer were 74% less likely to be satisfied with the cervical cancer screening service as compared to those who had poor knowledge [AOR: 0.26,95% CI: (0.12–0.59), p-value = 0.001]. This was confirmed by the in-depth interview finding of a 42 years old client. *“She did not tell me everything in detail. She just explained it roughly. But I have been asking extensively because I am an educated person. So*,* they need to explain everything to us thoroughly and clearly. It would be helpful if she explained that well.”*

The odds of cervical cancer screening satisfaction were 6.43 times more likely among clients who had favorable attitude compared to those who had unfavorable attitude [AOR: 6.43,95% CI: (3.43–12.03), p-value = 0.000]. this is supported by the finding of in-depth interview of a 32 years old client. *“I am now satisfied. I was extremely afraid that any small bit of pain I had would be cervical cancer. I was frightened that I had cervical cancer every time I watched television and heard about cancer. But from today I am free and happy. That is why I want to recommend other peoples”.*

## Result for the qualitative strand

### Sociodemographic characteristics of participants of the qualitative wing

A total of 14 participants were involved in the qualitative wing. Eight of them participated in the IDI, while six respondents participated in the KII. Interviews were conducted with women receiving cervical cancer screening services, service providers and administrators.

Most (88%) of IDI participants were urban residents. All of them (100%) were orthodox religion followers. Three fourth of the IDI respondents were government employee by occupation, majority of the participants were belonged to age range of 30–34(37%) and 35–39(37%) with mean age of 35 years. Most (75%) of them had attended college. Table 9.

**Table 9:** Characteristics of IDI participants in Debre Markos town public health facilities, Northwest Ethiopia, 2022 (*n* = 8)

Participants code	Age	Residence	Religion	Occupation	Education	Marital status
Participant#7	36	Rural	Orthodox	Housewife	Secondary	Married
Participant#8	35	Urban	Orthodox	Government employee	Degree	Married
Participant#9	42	Urban	Orthodox	Government employee	Degree	Divorced
Participant#10	32	Urban	Orthodox	Merchant	Secondary	Married
Participant#11	33	Urban	Orthodox	Government employee	Degree	Married
Participant#12	38	Urban	Orthodox	Government employee	Degree	Married
Participant#13	33	Urban	Orthodox	Merchant	Diploma	Married
Participant#14	30	Urban	Orthodox	Government employee	Degree	Married

Half (50%) of the KII respondents were aged between 35 and 39 years. More than three fourth (83%) of participants were Midwife by profession. The service year of the KII participant professionals were 2–15 years. Table 10.

**Table 10:** Characteristics of KII participants in Debre Markos town public health facilities, Northwest Ethiopia, 2022 (*n* = 6)

Participants code	Age	Sex	Profession	Responsibility	Year of experience
Participant#1	39	Male	Midwifery	MCH coordinator	13 years
Participant#2	38	Female	Health officer	MCH coordinator	15 years
Participant#3	36	Female	Midwifery	Provider	13 years
Participant#4	26	Female	Midwifery	Provider	9 years
Participant#5	32	Male	Midwifery	Provider	12 years
Participant#6	26	Female	Midwifery	Provider	2 years

### Theme

A total of 8 IDI and 6 KII were conducted. Three major themes were emerged from the thematic analysis; facility related barriers, clients related barriers and facility related facilitators. Table 11.

**Table 11:** Theme, subtheme and codes identified from the thematic analysis of participants interviews on cervical screening satisfaction at Debre Markos town public health facilities, Northwest Ethiopia, 2023

Theme	Subtheme	Code
Facility related barriers	Infrastructure problem	• No toilet • Room inconvenience to privacy • No shower • Poor cleanliness of the room • The room is not easily accessible
	Shortage of trained man power	• No constant provider assigned to the unit • No time to give adequate counselling • Doing in more than one service unit at the same time
	Shortage of supply	• Materials are not enough in number
	Providers skill gap	• Poor counselling • HCP cannot give LEEP service • No training on updated protocols
	Providers attitude	• Some providers are not willing to give health education • Some providers not think LEEP service is important
	Unavailability of full service	• No LEEP service • No chemotherapy services • No separate service unit for HIV/AIDS positive women
	Absence of respectful care	• Privacy was not maintained • Providers don’t pay attention to clients • Some of the facilities staffs are not friendly to clients • Talking on phone while examining
	Leadership problem	• Managers are only fault finders • Managers have no awareness on the service • Managers do not give attention to the service unit • They don’t respond on time
	Long waiting time	• No health care providers in the service unit • looking for the room for a long time
Client related barriers	Attitude	• No awareness in the community • Fear of disease cross contamination
	Gender preference	• Female provider preference
	Shyness to show genitals	• Embarrassing to be screened naked
Facility related facilitators	Free service	• It is exempted service
	Presence of supportive partners	• ICAP is providing materials • Other NGOs are supporting us
	Monitoring and evaluation	• There is supervision • There is regular monitoring and evaluation • Mentoring

### Theme 1: facility related barriers

#### Infrastructure problem

The study revealed that both the providers and managers frequently highlighted the shortage of infrastructure as a major issue. Clients also expressed dissatisfaction with the health facilities, citing inconvenience in receiving services due to inadequate infrastructure. They mentioned that the rooms were stuffy and did not provide enough privacy.

One client said, *“The screening room is narrow and used for both counseling and examination. It would be better if these were conducted in separate rooms*,* preferably in a more private location. During my examination*,* everyone was talking with the professional*,* and it was not comfortable.” (*Participant #10, 32 years, client).

*Another client added*,* “The screening area is too narrow and not well hidden. Although there is a curtain*,* it’s not sufficient. It’s not convenient for having private conversations*,* especially if someone unexpectedly enters the area.”* (Participant #13, 33 years old, client).

A provider also pointed out, *“There is no shower and toilet here*,* which needs improvement for mothers who come for cervical cancer screening. Many of them feel ashamed as they mainly come for other health services. This hinders them from seeking cervical cancer screening confidently.” (*Participant #4, 26 years, provider).

#### Shortage of skilled man power

A shortage of skilled manpower was also highlighted. Health professionals mentioned that due to staff shortages, they are often assigned to multiple service units simultaneously, affecting the availability and quality of cervical cancer screening services.

One participant mentioned, “*Two midwives were trained for cervical cancer screening*,* but they are also responsible for other maternal health care services*,* making it difficult for them to allocate dedicated time for cervical cancer screening.”* (Participant #2, 38 years old, coordinator).

Another participant added, “*The shortage of trained manpower and high workload result in us being unable to provide good quality service or individualized counseling for cervical cancer screening*.” *(*Participant #*3*,* 36 years old*,* provider)*.

#### Unavailability of full service

Additionally, the study found that hospital-level facilities were unable to provide advanced services for cervical cancer treatment due to a lack of essential equipment, medications, and skilled providers.

A participant reaffirmed.” *But there is no chemotherapy. To give full cervical cancer screening service chemotherapy service should be available. We are referring suspicious cases to Addis Ababa and Gondar hospitals. To have chemotherapy and radiotherapy services they have been going there. There is no chemotherapy to provide the full quality service*”. (Participant #1, 39 years old, coordinator).

#### The service providers skill gap

They expressed that they lacked the skills to provide advanced services for precancerous lesions and were not adequately updated on changes in screening and treatment protocols.

For instance, a participant mentioned, “*We have a LEEP machine*,* but the trained professional is not performing the procedure*,* indicating a skill gap. It seems that the procedure is challenging*,* and the necessary skills may not have been acquired during training.”* (Participant #1, 39 years old, coordinator).

Another midwife said “*Some health professionals have good counselling skill to convince their clients*,* in contrast most of them are poor at counselling and teaching. So*,* improving health professionals counselling skill is essential*”. (Participant #5, 32 years old, provider)

#### Scarcity of resources

Providers reported a scarcity of materials and supplies used for cervical cancer screening and treatment of precancerous lesions, particularly with instruments like speculums and forceps. Participants mentioned” *There are a variety of equipment’s. But when you see them in abundance*,* they are not enough. For example*,* a speculum is not sufficient. So*,* it would be great if these are fulfilled. Things like brochures are also important*”. (Participant #6, 26 years old, provider)

### Absence of respectful care

Some clients have reported being mistreated by providers and other healthcare facility staff. One client complained about the unfriendly behavior of security and cleaning staff. A respondent complained as follows “*The hospital services are being ruined by the security and cleaning staff. Guards are very unfriendly. Even they can hit you. They are stubborn. I have been hurt by them”. (*Participant #*14*,* 30 years old*,* client)*

Another client also expressed her disappointment with the service provider by saying*” He was talking on the phone while he was examining me with the device. I don’t think he really screened me properly because he was talking on the phone while examining me”. (*Participant #*12*,* 38 years old*,* client)*

### Leadership problems

Service providers raised concerns about leadership problems affecting the delivery of high-quality service. They mentioned that management does not pay equal attention to the cervical cancer screening unit compared to other service units and is unresponsive to their concerns.

A service provider stated *“We have tried to inform these problems to the hospital administrator. Still nothing is improved. They don’t pay attention for this class as for others service units. I don’t understand why. They don’t have a problem with the evaluating and monitoring. but they don’t evaluate the good things*,* they are just a fault finder”. (*Participant #*4*,* 26 years old*,* provider)*

### Long waiting time

Long waiting times for screening services have led to client dissatisfaction. Participants suggested that waiting times could be reduced by improving service accessibility and offering screening services in other service units, particularly at STI and ART clinics. The findings were evident with the following quotes: -.

*“Long waiting times for screening services have led to client dissatisfaction. Participants suggested that waiting times could be reduced by improving service accessibility and offering screening services in other service units*,* particularly at STI and ART clinics.”* (Participant #*7*,* 36 years old*,* client)*.

#### Theme 2: client related barriers

Client-related barriers were the second theme identified from the thematic analysis. Within this theme, three subthemes were identified: gender preference, knowledge and attitude, and shyness towards showing genitals.

### Provider’s gender preference

The majority of participants reported that they prefer female providers to males. They said they would not be embarrassed to expose their genitals to a female provider because they are biologically identical.

For example, one participant mentioned, *“It is good that the provider was a female. I will not ashamed to expose my genital to female provider*,* because we are the same nature. I am glad that I found a woman provider. if I offered to choose*,* I would choose a woman provider”. (*Participant #*11*,*33 years old*,* client)*

Another respondent noted that she *“I prefer a female professional to be transparent*,* and examined freely. You can communicate freely because talking to another woman is not humiliating or frightening. This is my preference. I don’t know about others”. (*Participant #*I3*,* 33*,* years old*,* client)*

### Knowledge and attitude

According to the respondents’ opinions, the level of the community’s knowledge and attitude toward cervical cancer screening is not satisfactory. They suggested that community mobilization needs to be done in a variety of approaches. Here are some sample quotes: “*Women in rural areas should be aware of that. It is unfair to serve only those who arrived*,* so they should encourage and teach others especially the rural communities to do the same”* (Participant #9, 42 years old, client).

Another respondent said, *“I think it would be nice if the awareness was created for the people on the outside as it is being given to us here. Even though it is challenging to directly educate the people*,* it is still feasible to raise awareness through the media”.* (Participant #14, 29 years old, client).

### Shyness to expose genitals

Respondents revealed that some clients were embarrassed to expose their genitals during the screening procedures. A participant noted, *“Some clients are ashamed of showing their genitals”* (Participant #2, 38 years old, coordinator).

Another service provider respondent added, *“With our clients*,* there is a certain shyness to expose their genital area. It is our culture. It is nothing for us as health professionals*,* but it’s difficult for our clients to show their genitals. It scares to show anyone*,* and naturally*,* it is shameful to expose the genital area”* (Participant #5, 32 years old, provider).

#### Theme 3: Facility related facilitators

Healthcare facility-related facilitators of quality cervical cancer screening were another theme that emerged from the thematic analysis. Three subthemes were identified from the theme: the presence of supportive partners, free service, and managerial supervision, monitoring, and evaluation.

### Presence of supportive partners

Support from different stakeholders is essential to provide quality services in healthcare settings. Study participants testified to the enormous contribution of partners, particularly a nongovernmental organization called ICAP, in providing high-quality cervical cancer screening. Here are some quotes from the respondents: *“But most of the time I directly communicate with ICAP organization. They support us”* (Participant #1, 39 years old, coordinator).

Another MCH coordinator added, *“We get acetic acid and other materials from a nongovernmental organization called ICAP. They provide us”* (Participant #2, 38 years old, coordinator).

### Free service

Respondents noted that the free provision of the service helps all clients to have access to the service regardless of their ability to pay. *“It is a free service. The service is being provided in combination with the maternal health care services”* (Participant #2, 38 years old, coordinator).

### Supervision, monitoring and evaluatione

The study respondents stated that their performance is being supervised, monitored, and evaluated regularly by managers. *“Our performance is being evaluated every week”.* (Participant #6, 26 years old, provider)

Another participant mentioned that the monitoring and evaluation by managers is helping them to pay attention to the service. *“The work is being evaluated every 6 months; it can be every 3 months. This is a good opportunity. It makes us pay attention to the work”.* (Participant #2, 38 years old, coordinator)

## Discussion

Client or patient satisfaction with health care services is clinically significant for building trust and engagement in the program, adherence to treatment instructions, and client retention. Therefore, this study aimed to evaluate client satisfaction and the factors influencing it with regard to cervical cancer screening services at public health facilities in Debre Markos town, Northwest Ethiopia, for the year 2022/23. The study found that two-thirds of clients were satisfied with the cervical cancer screening services they received. Factors such as perceived waiting time, knowledge and attitude of clients, and the gender of health care providers were significantly associated with client satisfaction with cervical cancer screening services.

According to this study, 65% of clients were satisfied with the cervical cancer screening services they received, which is higher compared to a study in Southern Ethiopia (41%) [14]. The differences observed could be due to variations in the cutoff points used for categorizing clients as dissatisfied or satisfied. In the above study, two confirmatory questions were used in addition to the five Likert-scaled parameters, while we used only five Likert-scaled questions.

However, the proportion of cervical cancer screening service satisfaction in this study was lower compared to a previous study conducted in Malawi (100%) [11]. The discrepancy could be attributed to variations in the parameters used for satisfaction assessment. In our study, we used 12 items with a five-point Likert scale to measure satisfaction, whereas in the study mentioned above, they used a single question for satisfaction assessment. The inconsistency may also be due to variations in the study periods, as clients’ knowledge improves and their expectations grow over time.

Similarly, our result showed a low satisfaction compared to a study in Morocco (98.2%) [13]. The difference may be attributed to variations in the sociodemographic characteristics of the study participants. In the study in Morocco, 60% of the participants were uneducated and rural residents, while only 31% of our participants were rural residents and 36% were uneducated. It is scientifically proven that rural residents and uneducated clients have higher satisfaction compared to their counterparts [37, 38]. Moreover, the differences in measures used for satisfaction assessment and study settings could be factors contributing to the discrepancies.

Satisfaction with cervical cancer screening services was found to be significantly associated with waiting time. Clients who reported shorter waiting times were 4.77 times more likely to be satisfied with the service compared to those who perceived longer waiting times. This finding aligns with studies in Southern Ethiopia, Morocco, and Malawi [11, 13, 14]. The possible explanation could be the fact that Clients often want quick service to return to their daily activities without wasting too much time.

This was supported by the result obtained from the in-depth interview of client participants which that they experienced long wait times for screening due to various reasons such as absence of health professionals, difficulty in accessing the screening unit, and lengthy registration processes. Providers also reported that clients faced long wait times due to the inaccessibility of the service in other units and a shortage of skilled staff.

Which was mentioned by a 36 years old woman as follows; *“Upon finding the room*,* I did not find anyone inside. There was no one in the screening room. There were no health professionals. So*,* I sat down and waited*,* then he came. I waited for at least 25 minutes. So*,* they shouldn’t disappear from their workplace”.*

The findings of the quantitative wing revealed that clients received CCSS from female health care providers were 6.11 times more satisfied with the service as compared to those who served by male health care providers. It is supported by findings from different studies [39–41]. This may be mainly for privacy purposes, as female providers are very aware of women’s physical and mental needs, clients feel comfort and communicate freely with female providers than male providers.

In agreement with the quantitative result, majority of clients in our qualitative strand noted that they prefer female provider to male. Additionally, key informant respondents recommended assigning female service providers to cervical cancer screening units as clients prefer female providers. The major reason for their preference is the fact that they value privacy and freedom over everything else. Clients interviewed revealed that they felt embarrassed and uncomfortable when examined by a male provider.

A 33 years old interviewed client said: - *“It is good that the provider was a female. I will not be ashamed of exposing my genitals to female health professionals*,* because we are the same nature. I am glad that I found a female provider. If I offered to choose*,* I would choose a female provider”.* Another a 32 years old client added *“I prefer a female professional to be transparent*,* and examined freely. You can communicate freely because talking to another woman is not humiliating or frightening. This is my preference. I don’t know about others”.*

Furthermore, satisfaction with CCSS was negatively correlated with clients’ knowledge of cervical cancer. Clients who had good knowledge were 74% less likely to be satisfied with the VIA screening service as compared to those who had poor knowledge. This is similar with studies conducted in Southern Ethiopia and Malawi [11, 14]. A possible explanation is that clients with good knowledge expect better service. If the actual service provided is not in balance with their expectations, it will end up with dissatisfaction.

The evidences from the qualitative wing also supports this finding. Most of highly educated and health professional clients were not satisfied with the information they got from health professionals regarding cervical cancer screening and privacy issues. Their complaints were that the information was unclear and the screening room was inconvenient for privacy. A 42-year-old client participant expressed disappointment with the explanations she received from her provider about cervical cancer and screening, as follows: - *“She did not tell me everything in detail. She just explained it roughly. But I have been asking extensively because I am an educated person. So*,* they need to explain everything to us thoroughly and clearly. It would be helpful if she explained that well.”*

Odds of reporting satisfaction were 6.43 times higher among women who had favorable attitude of cervical cancer as compared to those who had unfavorable attitude. This is in agreement with a study conducted in Thailand [12]. The explanation could be that, clients with positive attitudes are satisfied with the service because they only see the positive aspects. This result was confirmed by our qualitative findings of client respondents.

A 32-year-old interviewed client expressed satisfaction with having a cervical cancer screening service because she was concerned that she might have cervical cancer. *“I am now satisfied. I was extremely afraid that any small bit of pain I had would be cervical cancer. I was frightened that I had cervical cancer every time I watched television and heard about cancer. But from today I am free and happy. That is why I want to recommend other peoples”.*

## Limitation

Social desirability bias may have affected the findings of the study, as the client interviews were conducted on the premises of the health facilities. To minimize this bias, we employed non-staff data collectors and conducted the interviews with clients in a private area away from the screening units.

## Conclusion

According to the findings of this study, overall, two-thirds of clients were satisfied with cervical cancer screening services, which was lower than the national target of 80%. The study also identified modifiable facility and client-related factors affecting client satisfaction with cervical cancer screening services. Significant factors included waiting time, gender of service provider, and the attitudes and knowledge of clients. Additionally, qualitative responses indicated that shortage of infrastructure and resources, low skilled manpower, leadership-related problems, and healthcare professionals’ attitudes were among the top reasons for clients’ dissatisfaction with cervical cancer screening services.

## Recommendation

To Regional Health Bureau and Zonal Health Department.

- It is better to train healthcare providers on cervical cancer screening, especially LEEP services, and improving provider counseling skills to provide high-quality cervical screening services.
- Ensure accessibility of the service, availability of adequate skilled manpower and screening materials, and infrastructure in the facilities.

To health facilities.

- It is better implement strategies to ensure client satisfaction, including motivating screening providers, regular monitoring and evaluation, and making the screening services available in other units.
- It is better to assign both female and male providers to cervical cancer screening unit.
- It is better to serve clients based on their gender preferences.

To health care providers.

- It is better to present at their work place during working hours.
- It is better to counsel the cervical cancer screening recipients adequately.

To researchers.

- It is better to conduct qualitative and quantitative research at both public and private healthcare institutions.

**Ethical consideration**.

Ethical clearance was obtained from the Ethical Review Committee of the School of Midwifery under formal delegation given from the Institutional Review Board of the University of Gondar with a reference number MIDW/30/2015 E.C. A formal letter of administrative approval was obtained from each selected health facility.

Oral informed consent was obtained from each study subject, and each respondent was informed about the objective of the study. For participants who are not able to read and write, the informed consent form document prepared in local language (Amharic) read for the participants in presence of third party (close family member) and Informed consent was obtained after the participants and the third party were fully understood.

Participants were involved in the study after complete consent obtained. Study participants who did not willing to participate in the study were not forced to do so. Privacy and confidentiality of study participants were maintained.

## Data Availability

All data supporting these findings are available at the corresponding author upon requesting.

## Abbreviations

AIDS: Acquired Immune Deficiency Syndrome
AOR: Adjusted Odds Ratio
CCS: Cervical Cancer Screening
CCSS: Cervical Cancer Screening Service
CI: Confidence Interval
COR: Crude Odds Ratio
CSCCSS: Clients Satisfaction with Cervical Cancer Screening Services
DNA: Deoxyribose Nucleic Acid
ETB: Ethiopian Birr
HCP: Health Care Provider
HIV: Human Immune Virus
HPV: Human Papilloma Virus
ICAP: International Compliance Assurance Program
ID: Interpretive Descriptive
IDIs: In Depth Interviews
IUCD: Intra Uterine Contraceptive Device
KIIs: Key Informants Interviews
LEEP: Loop Electrosurgical Excision Procedure
LMIC: Low- and Middle-Income Countries
MCH: Maternal and Child Health
MMR: Mixed Method Research
OCP: Oral Contraceptive Pills
SD: Standard Deviation
SDG: Sustainable Development Goal
STI: Sexual Transmitted Infections
VIA: Visual Inspection with Acetic acid
WHO: World Health Organization
