# Correction: Clients’ satisfaction with cervical cancer screening services and influencing factors at public health facilities in Debre Markos town, Northwest Ethiopia, 2022/23: a convergent parallel mixed method

**DOI:** 10.1186/s12905-024-03468-3

**Published:** 2024-11-23

**Authors:** Alemu Merga Hailu, Fisseha Yetwale Kassie, Beyene Sisay Damtew, Muhabaw Shumye Mihret

**Affiliations:** 1 Department of Midwifery, School of Nursing and Midwifery, Institute of Health Sciences, Wollega University, Nekemte, Ethiopia; 2 Department of Clinical Midwifery, School of Midwifery, College of Medicine and Health Sciences, University of Gondar, Gondar, Ethiopia; 3 Department of Midwifery, School of Nursing and Midwifery, College of Medicine and Health Sciences, Arsi University, Asella, Ethiopia

**Correction: BMC Women's Health 24, 441 (2024)**

**https://doi.org/10.1186/s12905-024–03250-5**

Following publication of the original article [1], the author would like to include missing last names in the author group.

The author group should appear as mentioned below:

Alemu Merga Hailu^1*^, Fisseha Yetwale Kassie^2^, Beyene Sisay Damtew^3^ and Muhabaw Shumye Mihret^2^

The original article has been corrected.
